# Comparative colloidal stability, antitumor efficacy, and immunosuppressive effect of commercial paclitaxel nanoformulations

**DOI:** 10.1186/s12951-021-00946-w

**Published:** 2021-07-05

**Authors:** Jun Ye, Renjie Li, Yanfang Yang, Wujun Dong, Yujie Wang, Hongliang Wang, Tong Sun, Lin Li, Qiqi Shen, Caiyun Qin, Xiaoyan Xu, Hengfeng Liao, Yiqun Jin, Xuejun Xia, Yuling Liu

**Affiliations:** 1 State Key Laboratory of Bioactive Substance and Function of Natural Medicines, Institute of Materia Medica, Chinese Academy of Medical Sciences and Peking Union Medical College, 1 Xiannongtan Street, Beijing, 100050 People’s Republic of China; 2 Beijing Key Laboratory of Drug Delivery Technology and Novel Formulation, Institute of Materia Medica, Chinese Academy of Medical Sciences and Peking Union Medical College, Beijing, 100050 People’s Republic of China; 3 Beijing Wehand-Bio Pharmaceutical Co. Ltd., Beijing, 102600 People’s Republic of China

**Keywords:** Paclitaxel, Nanoparticle, Liposome, Emulsion, Stability, Immunosuppression

## Abstract

**Background:**

Standard chemotherapy with taxanes, such as paclitaxel (PTX), remains the mainstay of systemic treatment of triple-negative breast cancer. Nanotechnology-based formulations have gradually replaced PTX injection and are widely used in China. However, no studies have compared the colloidal stability, antitumor efficacy, and safety of commercial PTX nanoformulations. Additionally, the desire to evaluate preclinical antitumor efficacy in human-derived tumor cells led to the widespread application of immunodeficient mouse models that likely contributed to the neglect of nanomedicines-immune system interactions. The present study investigated the colloidal stability, antitumor efficacy and safety, and nanomedicines-host immune system interactions of PTX nanoformulations. A further comparative analysis was performed to evaluate the clinical potential.

**Results:**

Compared with liposome, PTX emulsion and PTX nanoparticle exhibited favorable colloidal stability. PTX emulsion was superior in inducing apoptosis and had a more pronounced inhibitory effect on 4T1-tumor spheroids compared with PTX liposome and PTX nanoparticle. Although PTX emulsion exhibited superior in vitro antitumor effect, no significant differences in the in vivo antitumor efficacy were found among the three types of PTX nanoformulations in an immunocompetent orthotopic 4T1 murine triple-negative breast cancer model. All PTX nanoformulations at maximum tolerated dose (MTD) induced lymphopenia and immunosuppression, as evidenced by the reduction of T cell subpopulations and inhibition of the dendritic cells maturation.

**Conclusions:**

The MTD PTX nanomedicines-induced lymphopenia and immunosuppression may weaken the lymphocyte-mediated antitumor cellular immune response and partly account for the lack of differences in the in vivo antitumor outcomes of PTX nanoformulations. Understanding of what impacts PTX nanomedicines has on the immune system may be critical to improve the design and conduct of translational research of PTX nanomedicines in monotherapy or combination therapy with immunotherapy.

**Graphic abstract:**

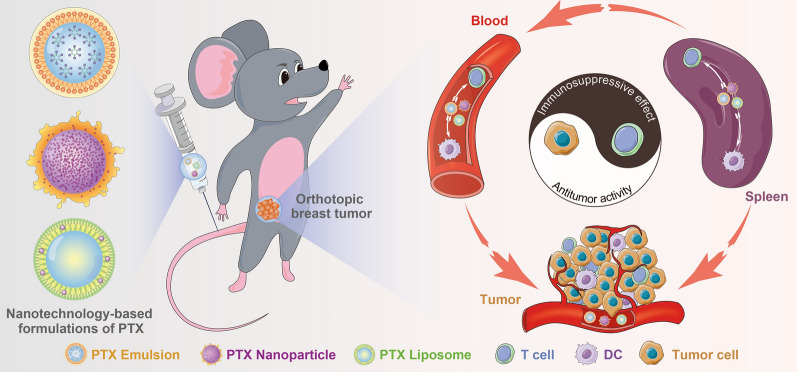

## Background

Breast cancer is the most commonly diagnosed cancer and the leading cause of cancer-related deaths in women [1]. Triple-negative breast cancer (TNBC) is the most malignant and aggressive subtype of breast cancer. TNBC is highly invasive, can often metastasize, is prone to relapse, and has a poor prognosis. Due to the lack of expression of the estrogen receptor, progesterone receptor, and human epidermal growth factor receptor 2, TNBC is not sensitive to endocrine therapy or molecular targeted therapy [2]. Chemotherapy is the established mainstay treatment for TNBC [3, 4]. Tumor-targeted delivery of chemotherapeutic agents has become a potential strategy to improve antitumor efficiency and attenuated chemotherapy-associated side effects [5].

Paclitaxel (PTX) is the most widely used drug in monotherapy or combination therapy for TNBC. The first commercial formulation, Taxol^®^, employed Cremophor EL and ethanol to enhance the solubility of PTX. Severe side effects limited its clinical application [6]. To reduce Taxol^®^-associated side effects and improve therapeutic efficacy, nanotechnology-based formulations of PTX, including liposome, emulsion, nanoparticle, and polymeric micelle, have been developed to permit the safe and efficient administration of PTX [5]. Lipusu^®^, formulation of injectable PTX liposome, which consists of PTX solubilized in 400 nm diameter liposome, was approved in China in 2003 as first-line chemotherapy for breast cancer. Although the absence of Cremophor EL, the clinical regimen of Lipusu^®^ employed a dose of 175 mg/m^2^ with corticosteroid premedication, presumably to match the commonly used regimen of Taxol^®^. In 2005, Abraxane^®^, a 130 nm albumin-bound nanoparticle form of PTX, was approved by the Food and Drug Administration (FDA) as second-line therapy for breast cancer without corticosteroid premedication [7]. The clinical results leading to approval of Abraxane^®^ demonstrated superiority relative to Taxol^®^ in terms of removal of corticosteroid premedication and reduction in toxicity, which in turn significantly enhanced the maximum tolerated dose (MTD; 175 mg/m^2^ vs. 260 mg/m^2^) [8]. Abraxane^®^ is one of the most prominent nanomedicine successful paradigms. This success has spurred the development of other nanoformulations of PTX to compete with Abraxane^®^, such as Cynviloq™, Paclical^®^, and PICN [7]. The predominant form of these commercial PTX nanoformulations is a lyophilized powder that is suspended in a specific vehicle solution just prior to clinical use. The colloidal stability of reconstituted nanoparticles is an important parameter in evaluating the clinical potential of PTX nanoformulations [9]. In our previous study, a tumor-targeting lipid emulsion of PTX, resembling a low-density lipoprotein lipid structure, was developed and already approved for clinical trials in China in 2019 [10]. The developed PTX emulsion dramatically enhanced the intracellular delivery of PTX into low-density lipoprotein receptor (LDLR)-overexpressing TNBC cells via LDLR-mediated endocytosis and exhibited superior safety and antitumor efficacy compared to Taxol^®^ at the MTD for TNBC treatment [11, 12]. Of note, compared with the commercial lyophilized powder of PTX nanoformulations, a distinctive advantage of PTX emulsion is the direct intravenous infusion that does not require any interventions.

FDA-approved nanomedicines reduce the toxicities associated with chemotherapeutic agents or solubilizers. Yet, their clinical use has thus far resulted in limited improvement in the overall survival of patients [13–16]. The rising use of cancer immunotherapy has reflected the increasing recognition of the critical roles of the immune system in both cancer progression and regression [17, 18]. However, the evaluations of preclinical antitumor efficacy in human-derived tumor cells led to the widespread application of immunodeficient mouse models that did not reveal nanomedicines-immune system interactions accurately [15]. Additionally, nanotechnology-based chemotherapy regimens continue to be applied at MTD, which inescapability induces drug resistance and systemic toxicity [19]. More importantly, such high dose regimens could result in lymphopenia and immunosuppression of host responses [20–23]. There is increasing evidence that the circulating lymphocytes play a central role in antitumor effect and chemotherapy-related lymphopenia and immunosuppression is associated with poor outcomes [21, 24, 25]. Thus, it is fair to question whether any key nanomedicines–host immune system interactions have been neglected that can account for the gap between the potential pharmacological advantages in preclinical studies and the limited improvement of clinical therapeutic efficacy [15, 16].

**Scheme 1:**
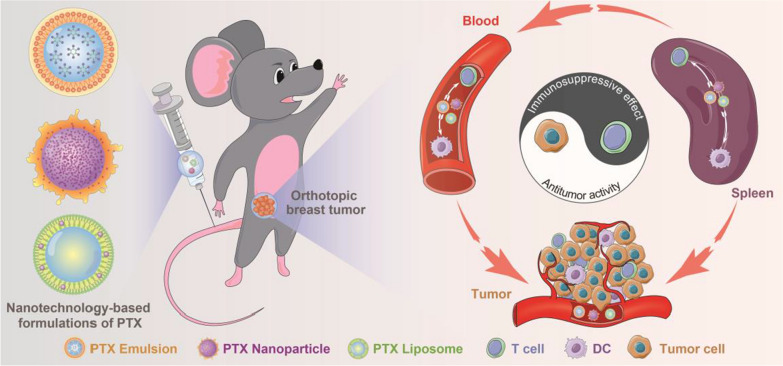
Schematic illustration of the interactions between PTX nanomedicines and host immune system in an immunocompetent 4T1 breast tumor-bearing mouse model

Although immunotherapy has potential therapeutic value in the clinical treatment of some types of cancer, the therapeutic effects on different TNBC subtypes are uncertain. Further, studies are required to confirm the efficacy and establish the best protocols for clinical practice [2, 17, 18]. Chemotherapy remains the established mainstay treatment for TNBC [26]. Nanotechnology-based formulations of PTX, including PTX liposome and nanoparticle for injection, are widely used chemotherapeutic nanomedicines in China. Accumulated studies compared the antitumor effect of PTX nanoparticle (Abraxane^®^) or PTX liposome (Lipusu^®^) with that of PTX Injection (Taxol^®^): PTX nanoparticle exhibited increased antitumor efficacy and improved therapeutic index in multiple human tumor xenograft models compared with an equitoxic dose of PTX injection; PTX liposome exhibited similar antitumor activity but its toxicity was lower than that of PTX injection [27–29]. However, no studies have compared the antitumor efficacy and safety of these commercial PTX nanoformulations head to head. Although the manufacturers prescribing information of PTX nanoparticle and PTX liposome indicated that reconstituted PTX nanoparticle or liposome in an infusion bag should be used immediately, but may be stable in the infusion bag at ambient temperature (approximately 25 °C) and lighting conditions for a maximum of 8 or 24 h. There have been no studies evaluating the in vitro colloidal stability of reconstituted nanoparticles and providing concrete stable information. Additionally, given the increasingly important effect of the immune system on the antitumor efficacy of chemotherapy, the interactions between nanomedicines and the host immune system that can occur using the MTD regimen should be investigated in immunocompetent mouse models. Similar to the commercial PTX nanoformulations, the PTX emulsion developed by our group and subsequently approved for clinical trials, also displayed superior safety and antitumor efficacy compared to Taxol^®^ when used at MTD to treat TNBC [11, 12]. In the present study, in vitro stability, in vitro and in vivo antitumor efficacy and safety, and nanomedicines–host immune system interactions of these PTX nanoformulations were investigated (Scheme 1). A further comparative analysis was performed. To the best of our knowledge, this is the first study to compare the chemotherapeutic capabilities and immune changes induced by available PTX nanoformulations. A better understanding of the general interactions between nanomedicines and host immune system is critical to improving the design and conduct of translational research of nanomedicines or combination with immunotherapy.

## Materials and methods

### Materials

PTX injection (Zisu^®^) was a kind gift from Beijing Union Pharmaceutical Factory (Beijing, China). PTX liposome for injection (Lipusu^®^) was produced by Nanjing Luye Pharmaceutical Co., Ltd. (Nanjing, Jiangsu, China). PTX nanoparticle for injection (albumin bound) (Aiyue^®^) was produced by Jiangsu Hengrui Pharmaceutical Co., Ltd. (Lianyungang, Jiangsu, China). PTX emulsion for injection was generated as described in our previous study and produced by Beijing Wehand-Bio Pharmaceutical Co., Ltd. (Beijing, China). Dulbecco’s modified Eagle’s medium (DMEM) was purchased from Thermo Fisher Scientific Inc. (Waltham, MA, USA). Fetal bovine serum (FBS) was purchased from ExCell Biotech Co., Ltd. (Shanghai, China). Matrigel^®^ was obtained from Corning (Corning, NY, USA).

### Cell line and animals

The 4T1 murine TNBC line was a kind gift from Prof. Zhonggao Gao (Institute of Materia Medica, Peking Union Medical College, Beijing, China). 4T1 cells were cultured in DMEM supplemented with 10% FBS, 100 U/mL penicillin, and 100 μg/mL streptomycin, and maintained at 37 °C in a humidified chamber with 5% CO_2_.

Female BALB/c mice (6–8 weeks old) were purchased from Beijing Vital River Laboratory Animal Technology Co. Ltd. (Beijing, China). All procedures involving experimental animals were performed according to the guidelines approved by the Institutional Animal Care and Use Committee of Institute of Materia Medica, Chinese Academy of Medical Sciences and Peking Union Medical College.

### In vitro characterization of PTX nanoformulations

The particle size distribution, polydispersity index (PDI), and zeta potential of PTX nanoformulations were determined by dynamic light scattering using a NICOMP 380 ZLS particle sizer (PSS NICOMP, Santa Barbara, CA, USA) after appropriate dilution. Lyophilized powder of PTX nanoparticle or PTX liposome was dissolved in 20 mL normal saline or 10 mL 5% glucose solution, respectively, according to the manufacturer’s instructions. PTX emulsion was taken with sterile syringes and then diluted with a specific vehicle solvent. Each sample was transferred to a light scattering cell and analyzed at different time points (0, 2, 4, 8, 12, 24, and 48 h). Each sample was analyzed three times. Morphological examination of PTX nanoformulations was performed by transmission electron microscope (TEM) (JEM1200EX, JEOL, Tokyo, Japan). These nanoparticle suspensions were stained with 2% (w/v) phosphotungstic acid, placed on a carbon film, and observed by TEM.

### In vitro colloidal stability

The in vitro colloidal stability of reconstituted PTX nanoformulations in specific vehicle solutions at 25 °C was qualitatively determined using a Turbiscan Tower^®^ (Formulaction, L’Union, France) by multiple light scattering [30]. The reconstituted PTX nanoformulations were appropriately diluted and individually placed in a glass cell and scanned using a light source. The transmission intensity profiles as a function of the position were acquired through a scan. The variations in the average transmitted intensity (ΔT) and turbiscan stability index (TSI) calculated from the signal value of transmission light were used as the main parameters to evaluate the in vitro colloidal stability of PTX nanoformulations.

### In vitro induction of apoptosis

The effect of PTX nanoformulations on the apoptosis of 4T1 cells was quantitatively evaluated using the Annexin V-FITC Apoptosis Detection Kit (Dojindo Laboratories, Tokyo, Japan). Briefly, 4T1 cells were seeded into 12-well plates at a density of 1 × 10^5^ cells per well and cultured at 37 °C for 24 h. The cells were treated with PTX nanoformulations at a PTX concentration of 0.1 µg/mL. After incubation for 24 h, the cells were washed and stained with Annexin V-FITC and propidium iodide according to the manufacturer’s protocol. The number of apoptotic cells was measured by flow cytometry. For the qualitative test, 4T1 cells treated with PTX nanoformulations (0.1 µg/mL PTX) were washed and stained with calcein AM and ethidium homodimer-1 (Invitrogen™) according to the manufacturer's protocol. The apoptotic red fluorescence signal was observed by fluorescence microscope (IX51; Olympus, Tokyo, Japan).

### Growth inhibition of three-dimensional (3D) breast tumor spheroids

The inhibitory effect of PTX nanoformulations on breast tumor spheroid growth was evaluated qualitatively and quantitatively based on changes in tumor sphere morphology and volume. 3D multicellular tumor spheroids of 4T1 cells were developed using a liquid-overlay system according to the culture scheme illustrated in Fig. 5A; [12]. Briefly, 4T1 cells (1 × 10^4^) were dispersed in culture medium containing 2.5% Matrigel^®^, seeded in wells of a 96-well round-bottom plate (Corning), and centrifuged at 1000×*g* for 10 min. After 4 days, separate groups of tumor spheroids were treated with PTX nanoformulations at a PTX concentration of 1 µg/mL. The day of administration was day 0. The morphology of the 3D tumor spheroids was observed by microscope and captured by Q-Capture software on days 0, 1, 3, 5, and 7. The major (d_max_) and minor (d_min_) diameters of each spheroid were measured, and the volume of spheroids was calculated using the following formula: V = (π × d_max_ × d_min_)/6.

### In vivo antitumor efficacy

The orthotopic 4T1 murine TNBC model was used to compare the antitumor efficiency of various PTX formulations [31]. Briefly, female BALB/c mice (6–8 weeks old) were inoculated subcutaneously into the right mammary gland with 100 µL of 4T1 cell suspension (1 × 10^6^ 4T1 cells). Three days after inoculation, the mice were randomly divided into five groups (n = 5 per group): (1) normal saline as control group; (2) PTX injection (20 mg/kg); (3) PTX emulsion (45 mg/kg); (4) PTX liposome (45 mg/kg); (5) PTX nanoparticle (45 mg/kg). All PTX formulations were injected via the tail vein on days 0, 4, and 8. Body weight was monitored regularly every 2 days during the treatment. On day 11, all mice were sacrificed by cervical vertebra dislocation. The tumors were collected, weighed, and photographed. Apoptosis of tumor tissue was analyzed using the terminal deoxynucleotide transferase (TdT)-mediated dUTP nick-end labeling (TUNEL) assay. The collected tumors were fixed with 4% paraformaldehyde (PFA), cut into 5 μm thick slices, and stained with the TUNEL assay kit (Roche) according to the manufacturer’s protocol. The prepared slices were observed by confocal laser scanning microscopy (TCS SP8X; Leica Microsystems, Wetzlar, Germany).

Major organs, including the heart, liver, spleen, lung, and kidney, were collected, fixed in 4% PFA, and subjected to histopathological examination using hematoxylin and eosin (HE) staining. To evaluate the biochemical characterization of mouse blood after the administration of PTX nanoformulations, the plasma was collected to detect alkaline aminotransferase (ALT), aspartate phosphatase (AST), lactate dehydrogenase (LDH), blood urea nitrogen (BUN), and creatinine (CRE) to evaluate hepatic and renal functions using an automatic biochemical analyzer (Accute TBA-40FR; TOSHIBA, Kawasaki, Japan). The peripheral blood and spleen of mice were collected and further analyzed to evaluate the in vivo immunosuppressive effect of PTX nanoformulations, including hematological indices and the immunophenotype of lymphocyte subsets.

### In vivo immunosuppressive effects

The in vivo immunosuppressive effect of PTX nanoformulations was evaluated by analyzing the immunophenotype of lymphocytes. Lymphocytes, including dendritic cells (DCs) and T cells, infiltrated into the peripheral blood and spleen were quantitatively analyzed by flow cytometry. Fresh spleens were cut into small pieces and ground into single-cell suspensions. The single-cell suspensions of spleen and peripheral blood were washed and stained with fluorescein-conjugated antibodies (Biolegend, San Diego, CA, USA). Erythrocytes were lysed by red blood cell lysis buffer. The cell suspensions were filtered through 400-mesh sieves after two washes and analyzed by flow cytometry (NovoCyte D3010; AECA Biosciences, San Diego, CA, USA). For immunophenotype analysis of T cells, CD4+ and CD8+ T cell levels were calculated as a ratio of the entire CD3+ population. For DC maturation analysis, The DCs were stained with CD11c and MHC II, and the expression levels of MHC I, CD40, CD80, and CD86 on DCs were examined [32, 33].

### Statistical analysis

All data subjected to statistical analysis were obtained from at least three parallel experiments. Statistical analysis was performed by one-way ANOVA for multiple groups using GraphPad Prism version 7.00 for Windows (GraphPad Software, La Jolla, CA, USA). Statistical significance was set at p ≤ 0.05.

## Results

### In vitro characterization

**Fig. 1:**
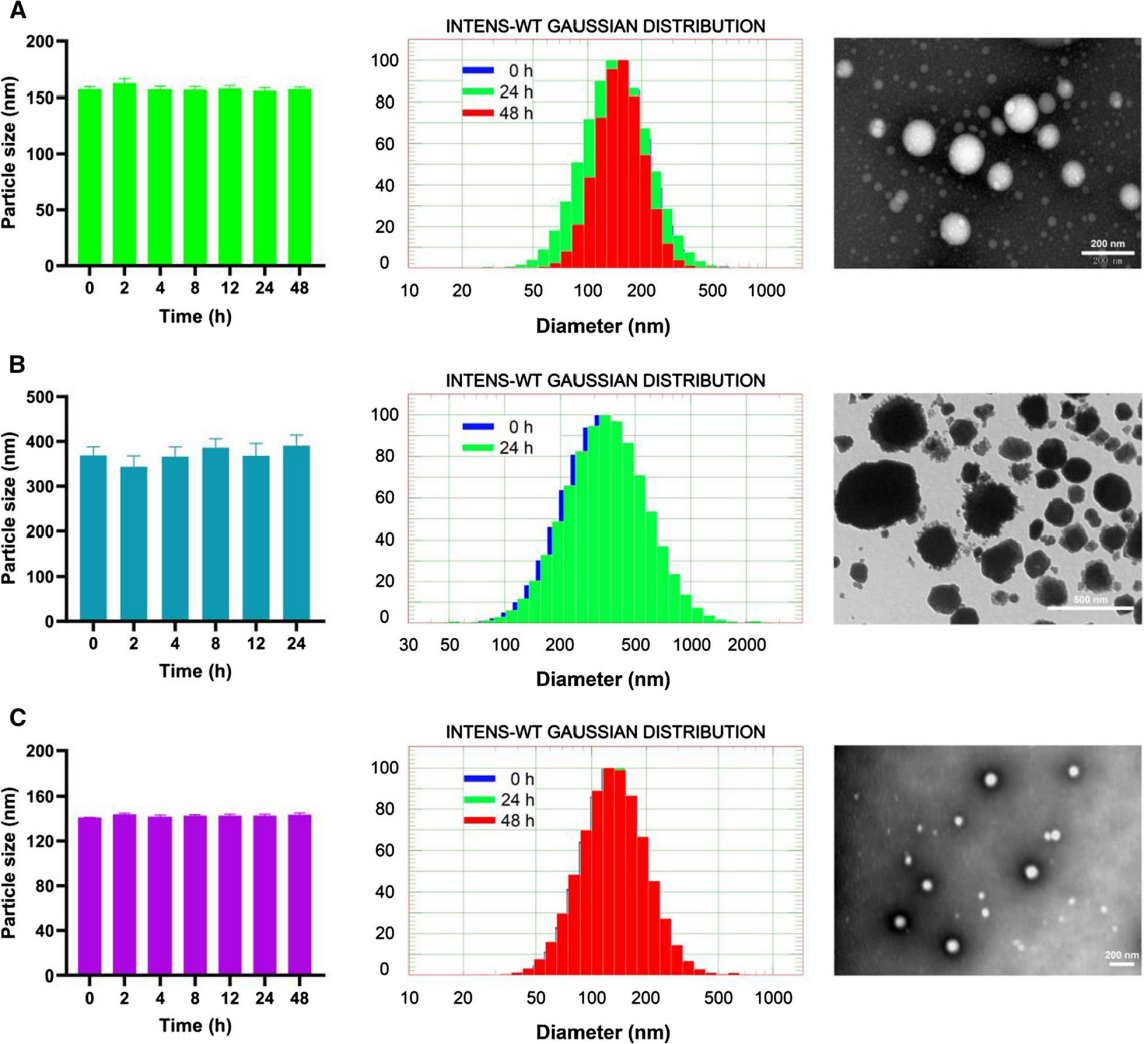
In vitro characterization of PTX nanoformulations. Mean particle size, particle size distribution, and TEM images of PTX emulsion (**A**), PTX liposome (**B**), and PTX nanoparticle (**C**). Each value represents the mean ± SD (n = 3)

**Fig. 2:**
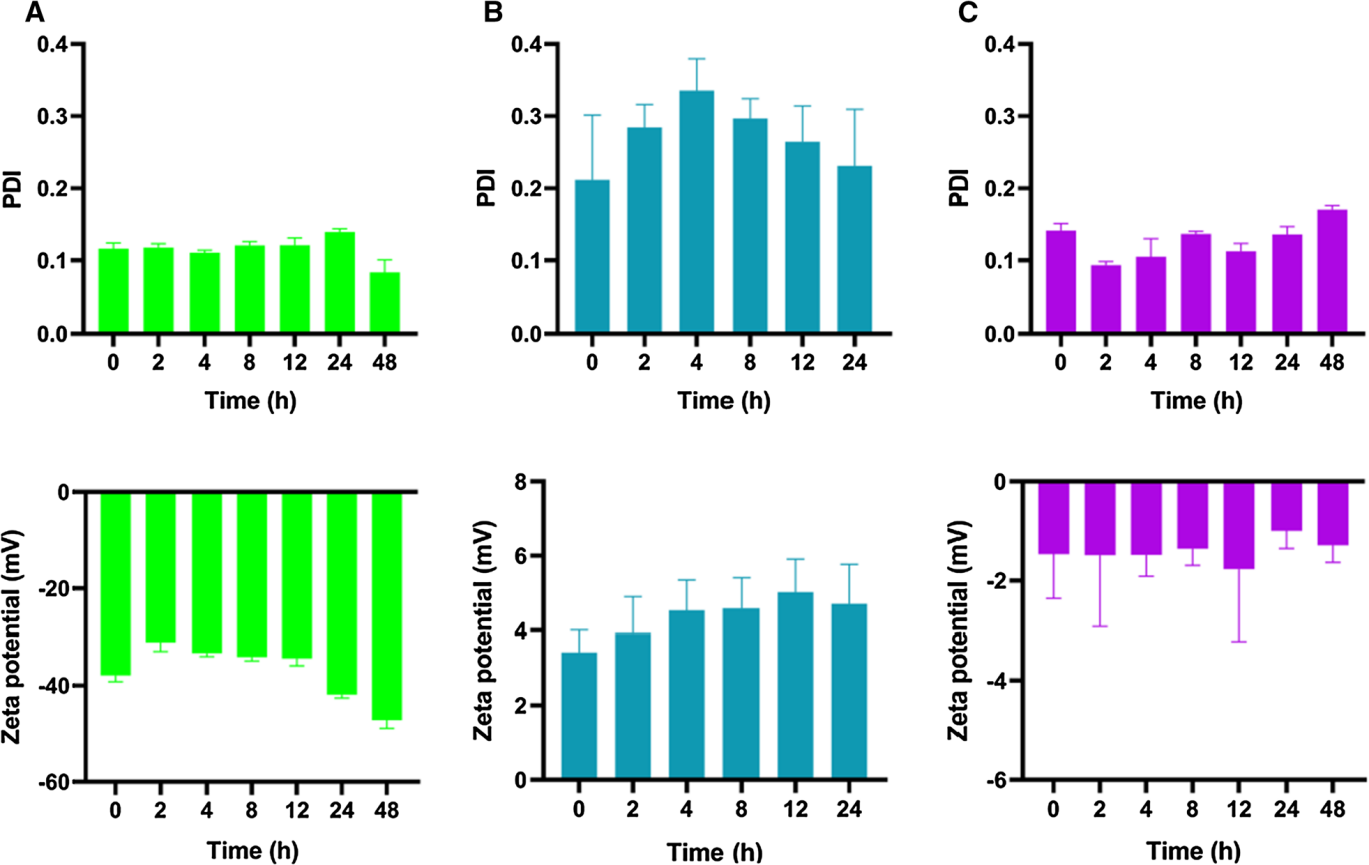
PDI and zeta potential of PTX emulsion (**A**), PTX liposome (**B**), and PTX nanoparticle (**C**). Each value represents the mean ± SD (n = 3)

The three PTX nanoformulations—PTX emulsion, PTX liposome (Lipusu^®^), and PTX nanoparticle (Aiyue^®^)—were characterized in terms of particle size distribution, zeta potential, and morphology. To investigate the properties of nanoformulations on temporal scales, the particle size distribution and zeta potential were monitored for 24 or 48 h. The mean particle size of freshly prepared PTX emulsion, PTX liposome, and PTX nanoparticle was 157.3 ± 2.0 nm, 368.8 ± 19.2 nm, and 141.1 ± 0.2 nm, respectively (Fig. 1). TEM revealed roughly spherical and subspherical morphology of the three freshly prepared nanoformulations. PTX emulsion and PTX nanoparticle displayed low PDI values (< 0.2), indicating narrow size distributions of these nanoformulations. The PDI of PTX liposome was slightly higher than that of the others, which could probably be attributed to its larger mean particle size. The zeta potential of freshly prepared PTX emulsion, PTX liposome, and PTX nanoparticle was − 30.9 ± 0.9 mV, + 23.6 ± 0.2 mV, and − 1.5 ± 0.9 mV, respectively (Fig. 2).

### In vitro colloidal stability

**Fig. 3:**
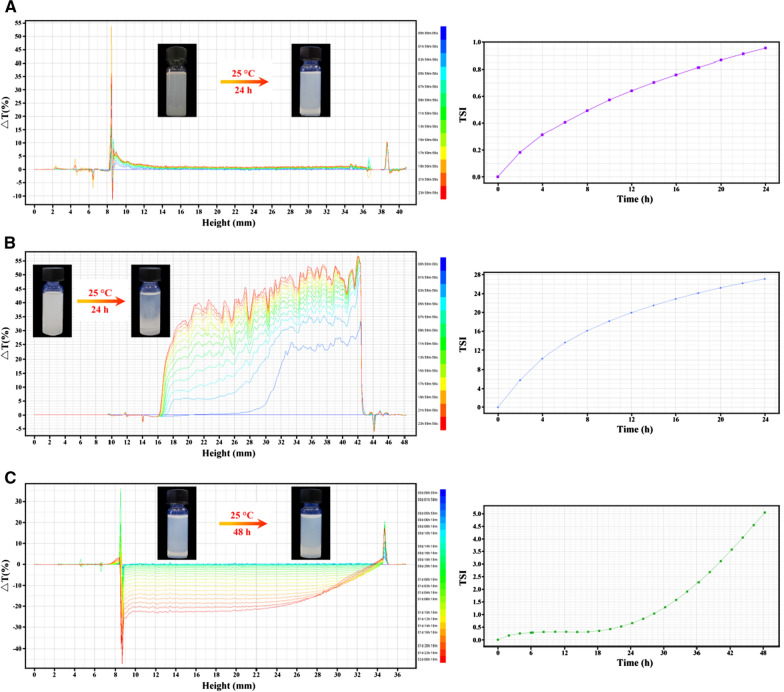
In vitro colloidal stability of PTX nanoformulations. Variations of transmission profiles (ΔT) and TSI of PTX emulsion (**A**), PTX liposome (**B**), and PTX nanoparticle (**C**), dispersed in a specific vehicle solution at 25 °C for 24 or 48 h

In terms of their colloidal stability against dispersion media, the mean particle size, PDI, and zeta potential of all the nanoformulations hardly changed within 24 h at 25 °C (Figs. 1, 2). To detect early imperceptible changes before the appearance of macroscopic physical modifications in nanoparticles, the colloidal stability of the nanoformulations was further assessed using the Turbiscan Lab^®^ Expert, an advanced analytical instrument. The variation in the droplet volume fraction or size was evident as a variation in the light transmission (ΔT) profiles. An unstable formulation was indicated when the variation was > 10% [30, 34]. As shown in Fig. 3, the variations in the transmission profiles (ΔT) of PTX emulsion and PTX nanoparticle were < 10%, indicating no apparent aggregation or sedimentation upon dispersion of these nanoformulations in dispersion media at 25 °C for 24 or 48 h. On the contrary, flocculation and sedimentation occurred when PTX liposome was dispersed in 5% glucose solution, as evidenced by the greater ΔT (about 50%) value and by their appearance after 24 h. TSI values were calculated from the changes in transmitted light, which were useful to compare the stability in more depth. Higher TSI values indicated lower stability [30, 34]. Although the TSI of all nanoformulations increased with time, PTX emulsion and PTX nanoparticle maintained relatively lower TSI values as compared with that of PTX liposome (Fig. 3). These results indicate that PTX emulsion and PTX nanoparticle possess favorable colloidal stability, which may be attributed to the surface modification of highly hydrated groups, such as poloxamer 188 or albumin. Additionally, the highly negative zeta potential of PTX emulsion was also responsible for greater stability.

### In vitro induction of apoptosis

**Fig. 4:**
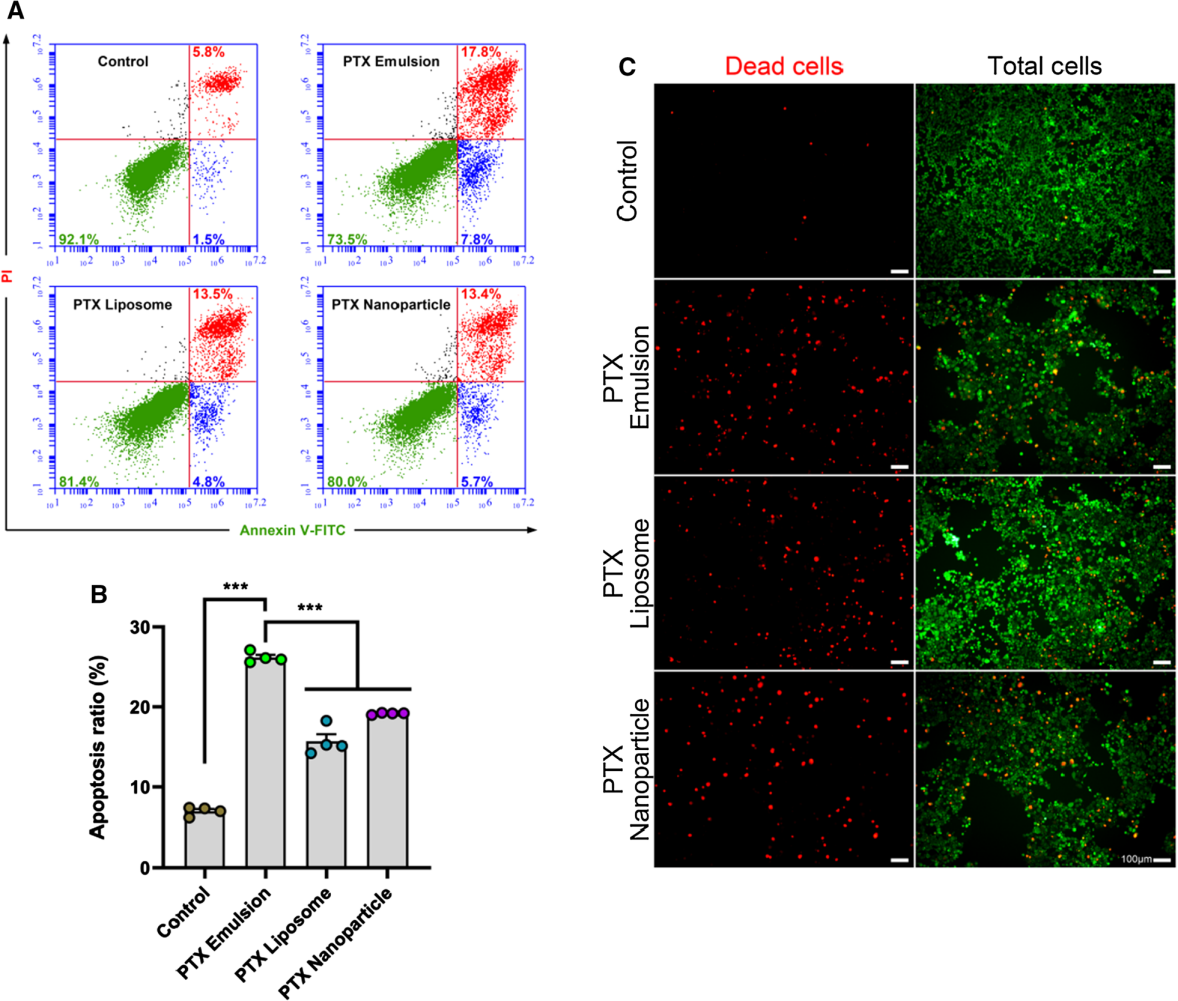
In vitro apoptosis-inducing effects of PTX nanoformulations in 4T1 cells. Proportion of apoptosis induced by PTX nanoformulations in 4T1 cells analyzed by flow cytometry (**A**, **B**) and fluorescence microscope (**C**). Red and green signal represent apoptotic cell and viable cell, respectively. ***p < 0.001. Each value represents the mean ± SEM (n = 4)

The Annexin V-FITC Apoptosis Detection Kit was used to quantitatively determine the apoptosis-inducing effects of all PTX nanoformulations in 4T1 cells. After treatment with cell culture, PTX emulsion, PTX liposome, and PTX nanoparticle, the percentages of apoptotic cells in 4T1 cells were 7.0 ± 0.6%, 26.2 ± 0.7%, 15.7 ± 1.8%, and 19.2 ± 0.1%, respectively (Fig. 4A, B). Compared with PTX liposome and PTX nanoparticle, PTX emulsion induced a significantly greater percentage of apoptotic 4T1 cells. The superior induction of apoptosis by PTX emulsion was also observed in the qualitative apoptosis analysis, in which more apparently apoptotic cells (red signal in Fig. 4C) were observed in comparison with those of PTX liposome and PTX nanoparticle. The increased apoptosis using PTX emulsion may be attributed to the targeted cellular uptake of PTX emulsion in 4T1 cells through the LDLR-mediated internalization pathway [11, 12].

### Inhibition growth of 3D tumor spheroids

**Fig. 5:**
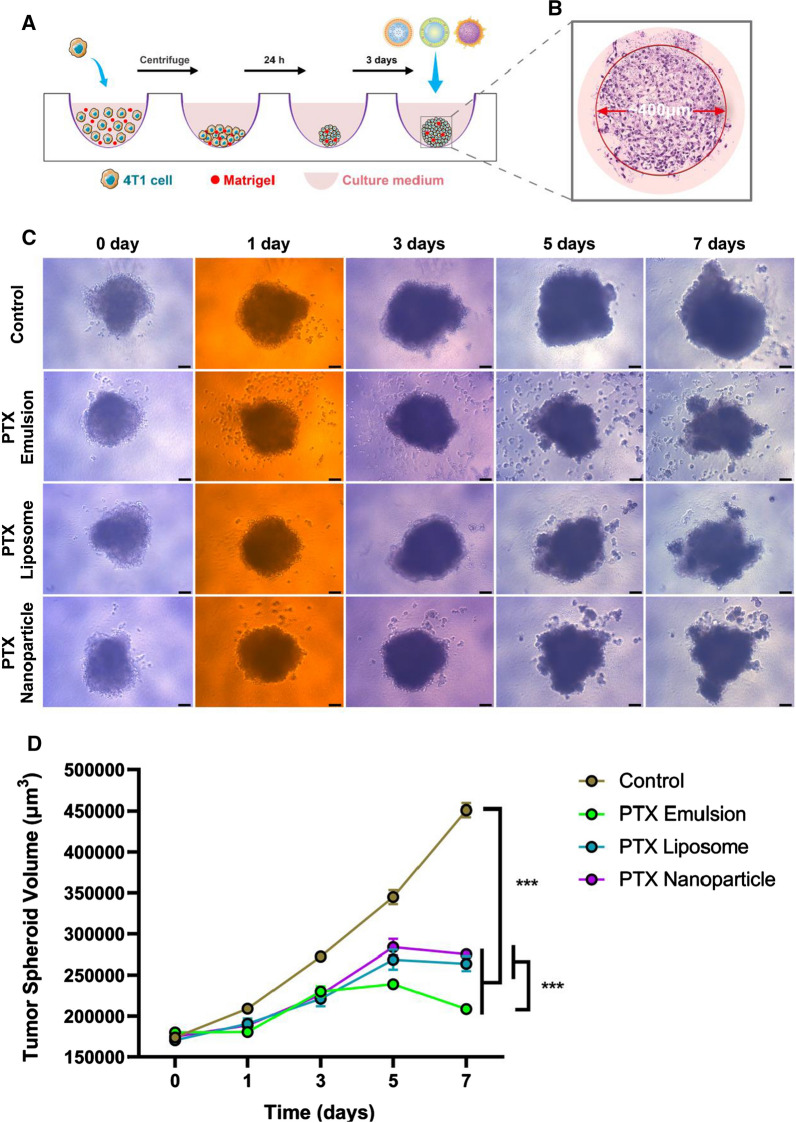
The inhibitory effect of PTX nanoformulations on 3D breast tumor spheroid growth. **A** The culture and inhibitory experiment procedure of 3D breast tumor spheroids. **B** HE staining image of 3D breast tumor spheroids. The changes in 3D tumor spheroids morphology (**C**) and volume (**D**) following the treatment of PTX nanoformulations. Scale bar = 120 μm. ***p < 0.001. Each value represents the mean ± SEM (n = 15)

4T1-tumor spheroids grew rapidly and became compact with time when treated with the cell culture medium (Fig. 5C, D). This was consistent with the growth characteristics of TNBC in vivo. After treatment with the three PTX nanoformulations, tumor spheroids grew progressively but more slowly in comparison with the control group, indicating that all PTX nanoformulations inhibited spheroid cell proliferation to some extent. After 7 days of treatment, the tumor spheroids of all PTX nanoformulations had obviously shrunk, with some cell detachment from the tumor spheroids. On day 7, the tumor spheroid volume of the PTX emulsion-treated group was much lower than that of PTX liposome- and PTX nanoparticle-treated group. Cell detachment from the tumor spheroids was markedly more evident in the presence of PTX emulsion, and so was a much greater inhibition of 4T1-tumor spheroids. In our previous study, PTX emulsion penetrated more deeply and was distributed more extensively in tumor spheroids formed by TNBC cells than in non-TNBC cells, which may be attributed to the LDLR-mediated targeted delivery [12]. The higher penetration efficiency of PTX emulsion led to a much more pronounced inhibitory effect on 4T1-tumor spheroids, which was also consistent with the results of in vitro cell apoptosis described earlier.

### In vivo antitumor efficacy

To compare the in vivo antitumor efficiency of PTX nanoformulations on orthotopic 4T1 tumor-bearing mice, the mice were intravenously injected with PTX injection (20 mg/kg), PTX emulsion (45 mg/kg), PTX liposome (45 mg/kg), or PTX nanoparticle (45 mg/kg), respectively. As chemotherapy is generally administered at the highest tolerated dose, the comparison of MTD, rather than equal doses, is considered more clinically relevant [27]. In our previous study, we found that the MTD of PTX injection and PTX emulsion in healthy BALB/c nude mice was 20 and 45 mg/kg (2.25-fold), respectively, indicating that PTX emulsion was significantly less toxic than PTX injection [11]. Similar to the results of PTX emulsion, the MTD of PTX nanoparticle was 2.24-fold higher than that of PTX injection [27]. Based on these results, the doses of 20 mg/kg and 45 mg/kg were chosen for PTX injection and PTX nanoformulations, respectively, for the in vivo antitumor efficacy study.

**Fig. 6:**
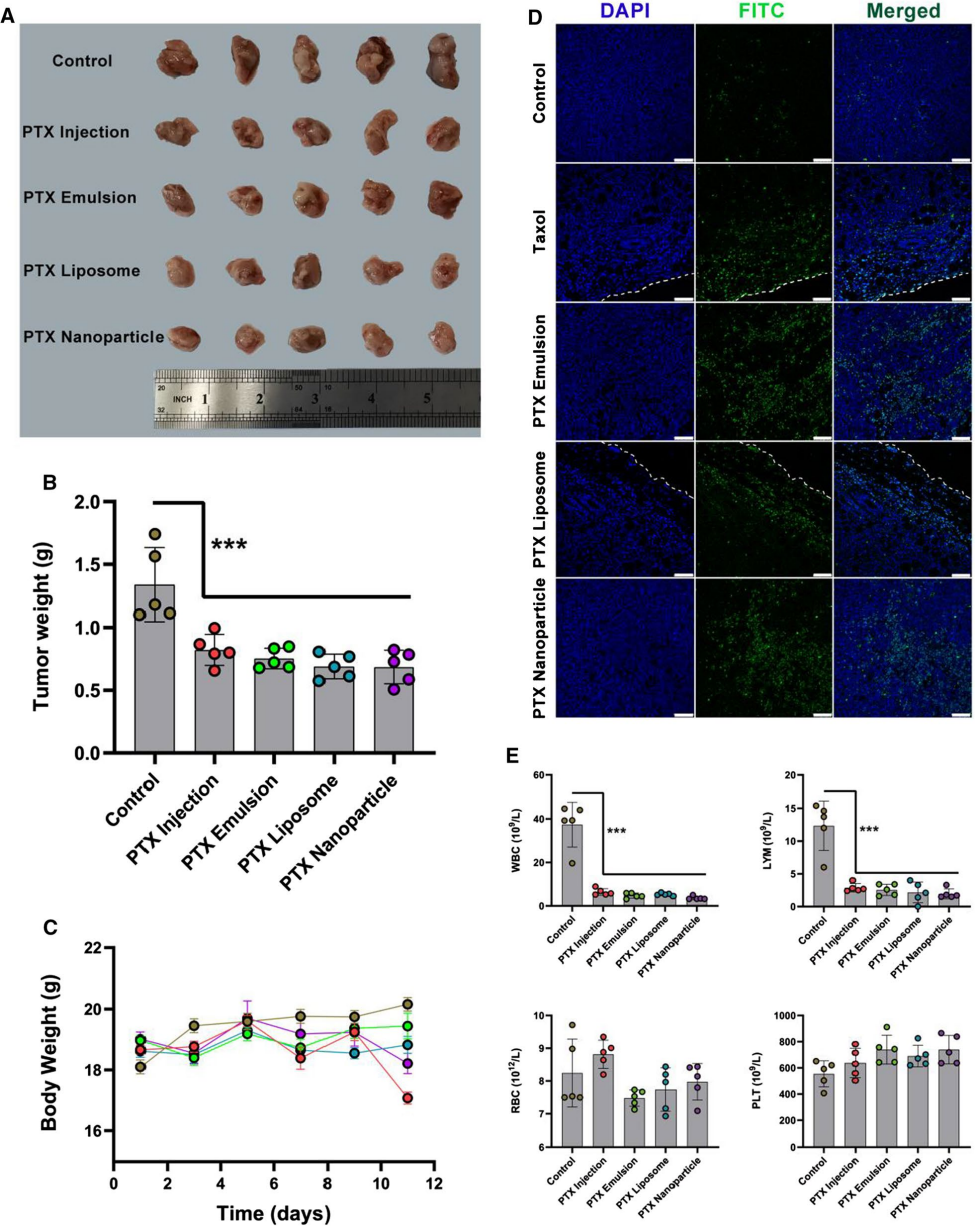
In vivo antitumor efficacy of PTX nanoformulations in an orthotopic 4T1 murine TNBC model. The images of excised tumors (**A**), tumor weight (**B**), and body weight changes (**C**) of 4T1 breast tumor-bearing mice after treatment of PTX nanoformulations. **D** TUNEL staining images of excised tumors. **E** The hematological examination, including the level of WBC, RBC, PLT, and LYM. Scale bar = 75 μm. Each value represents the mean ± SEM (n = 5). ***p < 0.001

As expected, PTX injection and the three types of PTX nanoformulations produced significant antitumor efficacy against 4T1 tumors, as evidenced by the decreased tumor volume and tumor weight compared with those of the control group (Fig. 6A, B). However, there was no significant difference in the antitumor efficacy between the PTX injection and the PTX nanoformulations, as well as among PTX emulsion, PTX liposome, and PTX nanoparticle. To further evaluate the therapeutic effects, the tumors were collected to assess tumor cell apoptosis among different groups using the TUNEL assay at the end of the experiment. As shown in Fig. 6D, there was a very faint apoptotic fluorescence signal (green) in the control group. Compared with the control group, the PTX injection- and all PTX nanoformulations-treated groups displayed markedly more intense apoptotic fluorescence signals, consistent with the decreased tumor weight. Interestingly, apoptosis in the PTX emulsion- and PTX nanoparticle-treated groups was primarily distributed in the center of the tumors, while that in the PTX injection- and PTX liposome-treated groups was observed in the periphery of the tumors. The different distribution profiles of the apoptotic cases in these treatment groups may be attributed to the tumor penetration ability of PTX emulsion and PTX nanoparticle: PTX emulsion facilitates intracellular delivery of PTX into breast cancer cells via LDLR-mediated endocytosis and PTX nanoparticle exploits the natural property of albumin to reversibly bind to PTX, transports it across the endothelial cell via glycoprotein 60-mediated transcytosis, and accumulates it in tumors by albumin binding to SPARC (secreted protein, acidic and rich in cysteine) [12, 35–37].

### Evaluation of safety in vivo

**Fig. 7:**
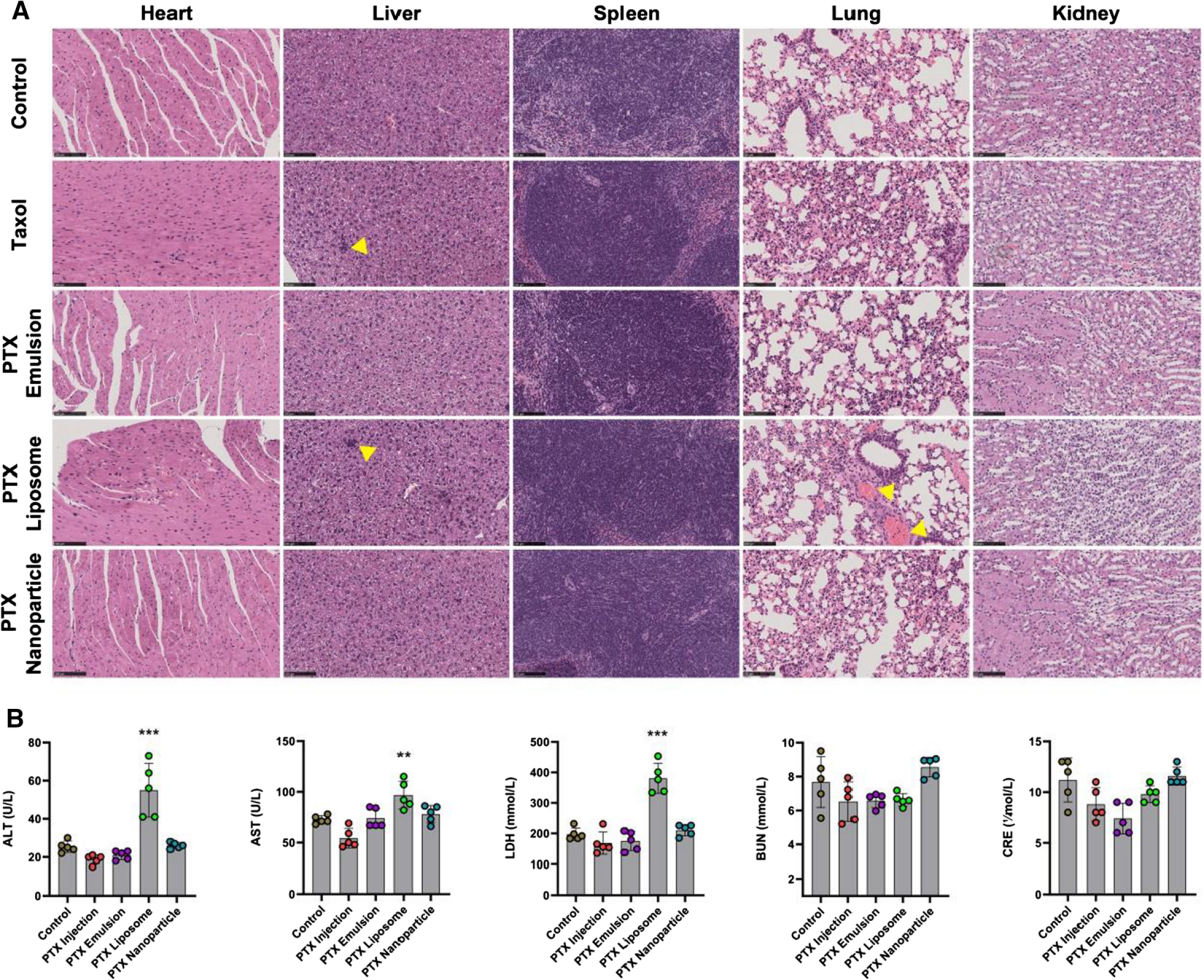
In vivo safety evaluation of PTX nanoformulations. **A** Histopathologic analyses of HE-stained tissue sections from heart, liver, spleen, lung, and kidney after the indicated treatment. Scale bar = 100 μm. Yellow triangular arrows represent the pathological changes. **B** The determination of blood biochemistry parameters including ALT, AST, LDH, BUN, and CRE levels. Each value represents the mean ± SEM (n = 5). **p < 0.01 and ***p < 0.001 compared with the control group

The in vivo safety of PTX formulations was preliminarily assessed by observing animal behavior and body weight after administration. Abnormal behavior, such as lying down and decreased overall activity, was observed in mice treated with PTX injection, which was likely due to a hypersensitivity reaction to Cremopher EL. In contrast, mice treated with PTX nanoformulations tolerated the regimens well. As shown in Fig. 6C, mice treated with PTX injection exhibited slight body weight loss. There was no obvious body weight loss in the mice treated with PTX nanoformulations. The safety of PTX formulations in the main organs was further evaluated by histopathological examination. As shown in Fig. 7A, no obvious organ lesions (heart, liver, spleen, lung, and kidney) were observed in both the PTX emulsion- and PTX nanoparticle-treated groups as compared with the control group. However, mice treated with PTX injection and PTX liposome exhibited a certain degree of fragmentation and pathological changes in the liver and lung. As shown in Fig. 7B, the elevated level of plasma ALT, AST, and LDH in the PTX liposome-treated group, indicating that PTX liposome induced liver and lung injury in mice [38]. PTX emulsion and PTX nanoparticle were safe, likely due to their targeted delivery and associated enhanced tumor accumulation.

### In vivo immunosuppression

In addition to its wide-ranging adverse effects on non-target tissues, cytotoxic chemotherapy is typically immunosuppressive because of its toxicity to dividing cells in the bone marrow and peripheral lymphoid tissues. Lymphopenia is considered the most serious immunosuppressive toxicity [20–23]. Previous studies reported the effects of chemotherapy on lymphocytes in breast cancer patients, with a consensus that chemotherapy induces a progressive decline in all T-lymphocyte subpopulations [39–41]. To evaluate the immunosuppressive toxicity of PTX formulations, peripheral blood was collected to determine the hematological indices, including the level of white blood cells (WBC), red blood cells (RBC), platelets (PLT), and lymphocytes (LYM) among different groups using an automatic hematology analyzer at the end of the experiment. As shown in Fig. 6E, PTX formulations had no significant effect on the levels of RBC and PLT. However, the total WBC and level of LYM in the peripheral blood were dramatically reduced by treatment with all PTX formulations, indicating that PTX formulations at the MTD, including nanoformulations, could significantly decrease the circulating lymphocyte levels, which is consistent with previous studies [39–41]. Lymphocytes, with T cells, B cells, and dendritic cells (DCs) as the typical representatives, are a very important immune population involved in the antitumor immune response. Therefore, the reduction of circulating lymphocyte levels induced by PTX formulations may impair the antitumor activity of the immune system.

**Fig. 8:**
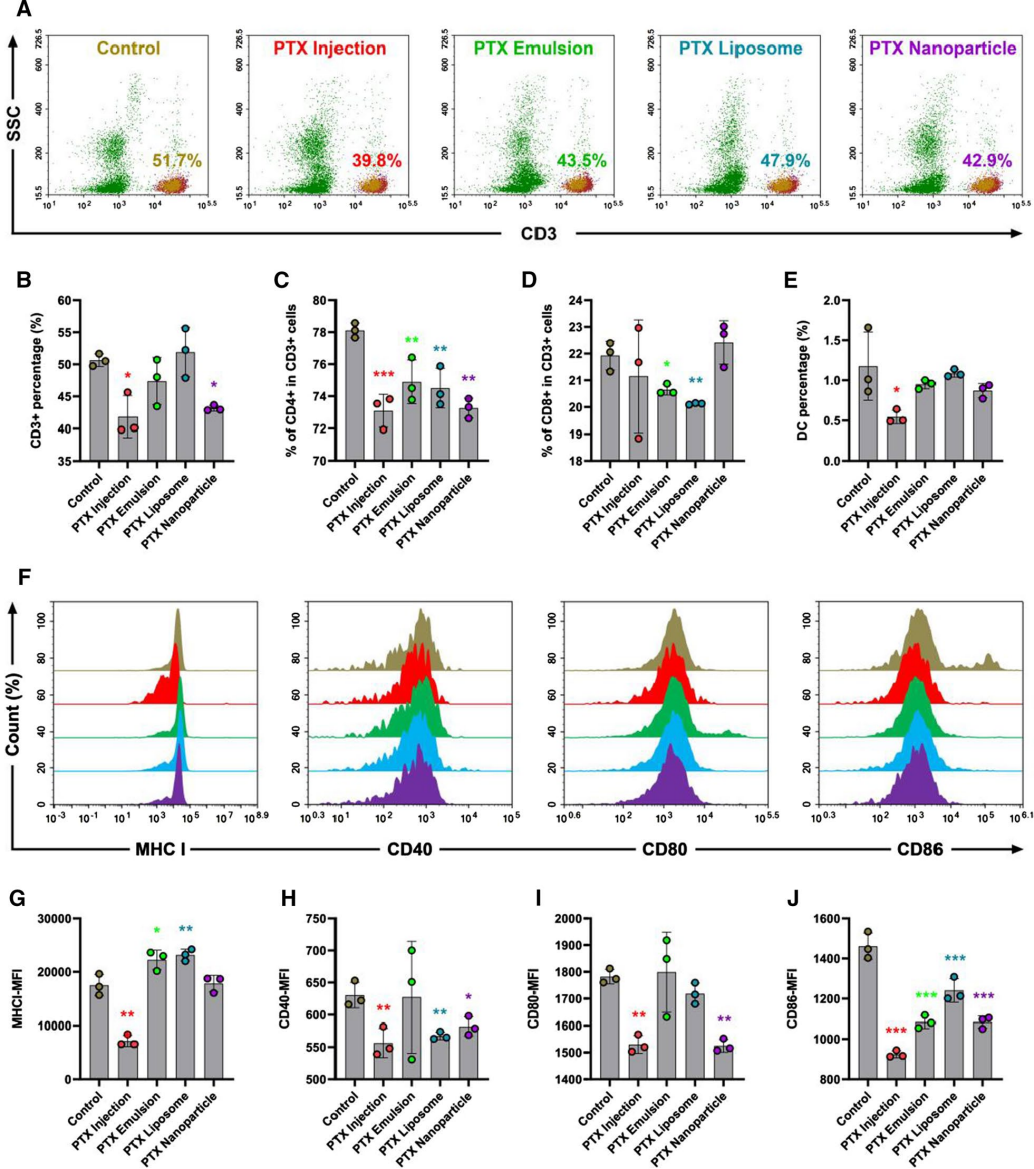
The immunosuppressive effect of PTX nanoformulations on lymphocytes in peripheral blood. **A** Representative flow cytometry analysis of CD3+ T cells. **B**–**E** The percentage of CD3+, CD4+ in CD3+ cells, CD8+ in CD3+ cells, and DCs. **F**–**J** Flow cytometer analysis for the expression of DCs maturation markers: MHC I, CD40, CD80, and CD86 of DCs in peripheral blood. The DCs were stained with CD11c and MHC II. Each value represents the mean ± SEM (n = 3). *p < 0.05, **p < 0.01, and ***p < 0.001 compared with the control group

To further evaluate the immunosuppressive effect of PTX formulations on lymphocytes, the percentages of lymphocyte subsets (T cells and DCs) in peripheral blood were determined by flow cytometry. As shown in Fig. 8A–E, PTX emulsion and PTX liposome had no significant effect on the percentage of CD3+ T cells, while significant depletion of CD3+ T cells was induced by PTX injection and PTX nanoparticle as compared to the control group. All mice treated with PTX formulations experienced a dramatic decrease in the percentage of CD4+ in CD3+ cells, and both PTX emulsion and PTX liposome also decreased the percentage of CD8+ in CD3+ cells. Similarly, the proportion of DCs was also reduced to some extent after treatment with PTX formulations, with PTX injection exerting the most pronounced reduction. The maturation of DCs is closely involved in their ability to prime naive T cells into effector T cells [42]. The expression of classical DC maturation markers (MHC I, CD40, CD80, and CD86) was determined to better understand the effects of chemotherapy on the immune function of T cells and DCs, including the maturation of DCs and subsequent activation of effector T cells. As shown in Fig. 8F–J, MHC I, CD40, CD80, and CD86 expression levels in the PTX injection group were significantly lower than those in the control group. PTX nanoparticle significantly decreased the expression levels of CD40, CD80, and CD86, and PTX liposome dramatically reduced the expression levels of CD40 and CD86. Only one type of classical DC maturation marker (CD86) was downregulated by the PTX emulsion. Water-in-oil emulsion has been applied as an effective adjuvant in vaccine formulation for many years [43]. Some commercial adjuvants based on emulsion technology, including MF59 and AS03, have been licensed and shown to improve antibody responses to antigen [44]. It is possible that PTX emulsion also has a certain immune adjuvant effect that neutralizes the inhibition effect of PTX on DC maturation. However, its underlying mechanism still needs further verification in our future study.

**Fig. 9:**
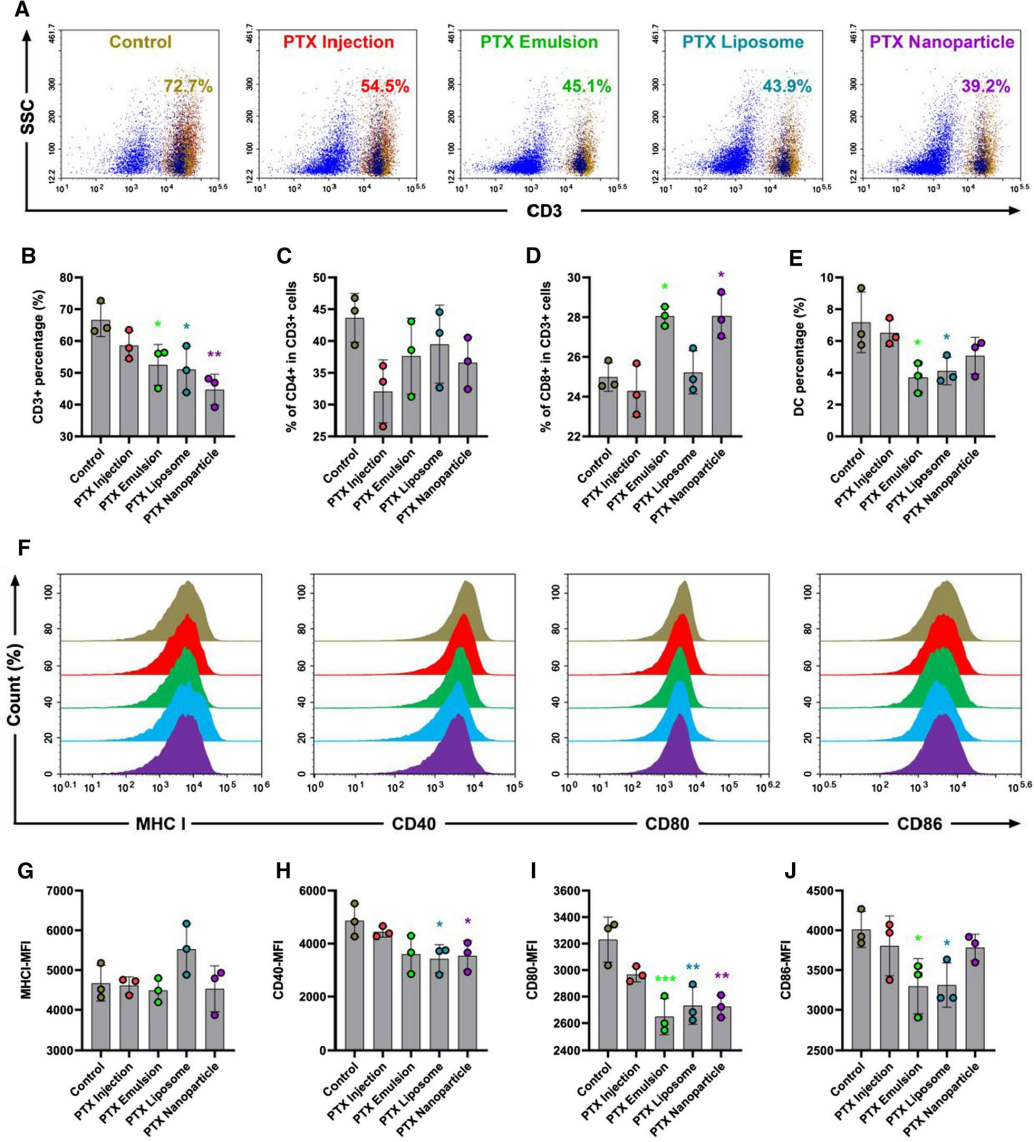
The immunosuppressive effect of PTX nanoformulations on lymphocytes in spleen. **A** Representative flow cytometry analysis of CD3+ T cells. **B**–**E** The percentage of CD3+, CD4+ in CD3+ cells, CD8+ in CD3+ cells, and DCs. **F**–**J** Flow cytometer analysis for the expression of DCs maturation markers: MHC I, CD40, CD80, and CD86 of DCs in spleen. The DCs were stained with CD11c and MHC II. Each value represents the mean ± SEM (n = 3). *p < 0.05, **p < 0.01, and ***p < 0.001 compared with the control group

The spleen is one of the most important immune organs involved in nonspecific and specific immunity, and is the source of lymphocytes. The percentages of lymphocyte subsets in the spleen are worth studying. In the spleen, PTX nanoformulations had a much greater effect on the depletion of T cells and DCs than PTX injection, which may be attributed to the enhanced accumulation of PTX nanocarriers in the organs of the reticuloendothelial system, such as the spleen. As shown in Fig. 9, three types of PTX nanoformulations significantly reduced the percentage of CD3+ T cells. Additionally, depletion of DCs was also observed in the PTX emulsion- and PTX liposome-treated groups. The PTX nanoparticle-treated group displayed a decreasing tendency in the percentage of DCs, but the difference was not statistically significant compared to that of the control group. In terms of the expression of classical DC maturation markers, PTX liposome exhibited the most pronounced effect, which simultaneously downregulated the expression levels of CD40, CD80, and CD86. There was a decrease in the expression of CD80 and CD86 in the PTX emulsion groups and only the expression of CD40 was decreased in the PTX nanoparticle group.

Overall, these data indicates that PTX formulations at MTD induce the reduction of T cell subpopulations and inhibit the maturation of DCs in both the peripheral blood and spleen of immunocompetent mice. Interestingly, PTX injection exhibited a more pronounced immunosuppressive effect in peripheral blood than PTX nanoformulations, whereas PTX nanoformulations had a more significant immunosuppressive effect in the spleen than PTX injection. This could be related to the ability of bare nanoparticles to accumulate in organs of the reticuloendothelial system, thereby promoting its immunosuppressive effect in the spleen.

## Discussion

Compared with other subtypes of breast cancer, TNBC is characterized by a worse prognosis, a higher risk of recurrence and metastasis, and shorter survival after recurrence. Standard chemotherapy with taxanes, such as PTX, is still the mainstay of systemic TNBC treatment [26]. Nanotechnology-based formulations of PTX, including PTX liposome and nanoparticle for injection, have gradually replaced PTX injection and are widely used in China. However, no studies have compared the in vitro colloidal stability, and the in vitro and in vivo antitumor efficacy and safety of the commercial PTX nanoformulations.

Nanoparticles are often prepared as aqueous suspensions. However, when suspended in an aqueous medium, nanoparticles are physically unstable and can aggregate, fuse, and sediment; and drug leakage can occur during long periods of storage [9]. To overcome these problems, most commercial nanotechnology-based formulations of PTX are lyophilized to produce a solid dosage form. The maintenance of the mean diameter and particle size distribution of nanoparticles after lyophilization is a key parameter for an acceptable lyophilized form of nanoformulation [9]. Additionally, the colloidal stability of lyophilized nanoparticles reconstituted in a specific injection medium directly affects the safety and efficacy of nanoformulations during clinical use. As shown in Fig. 1, the mean diameter and particle size distribution of lyophilized PTX nanoparticle and liposome freshly reconstituted in a specific injection medium, remained almost unchanged within 24 or 48 h. As expected, PTX emulsion that did not require reconstitution before clinical use also exhibited good colloidal stability based on the mean diameter and particle size distribution. To further characterize the in vitro colloidal stability of PTX nanoformulations, Turbiscan Lab^®^ Expert was used to precisely monitor the variation of the relative stability by transmitted light in TSI, which could detect early imperceptible changes before the appearance of macroscopic physical modifications in nanoparticles. As shown in Fig. 3, PTX emulsion and PTX nanoparticle exhibited good colloidal stability within 24 h, as evidenced by ΔT (< 10%) and TSI (< 1). However, flocculation and sedimentation occurred when PTX liposome was reconstituted in 5% glucose solution, as reflected by the higher ΔT (> 10%), TSI (> 20), and its appearance. Compared with the determination of mean diameter and particle size distribution, ΔT and TSI values obtained using the Turbiscan Lab^®^ Expert allow for a more objective and accurate evaluation of the colloidal stability of PTX nanoformulations. Samples needed to be shaken and mixed well before the mean diameter and particle size distribution measurements. Thus, the flocculated nanoparticles re-dispersed in the medium, and the mean diameter and particle size distribution hardly changed in a short time. In contrast, the Turbiscan Lab^®^ Expert could detect and visually display early imperceptible changes. The favorable colloidal stability of PTX emulsion and PTX nanoparticle may be attributed to the surface modification of highly hydrated groups, such as poloxamer 188 or albumin. Additionally, the highly negative zeta potential of the PTX emulsion was also responsible for its greater stability. The lower colloidal stability of PTX liposome indicated that intravenous infusion should be carried out as soon as possible after reconstitution, which significantly affects their safety and efficacy.

In addition to the colloidal stability, a comparative study of the in vitro and in vivo antitumor efficacy of PTX nanoformulations was performed to further evaluate the clinical potential. As shown in Figs. 4, 5, PTX emulsion was superior in inducing apoptosis of 4T1 cells, with a markedly pronounced inhibitory effect on 4T1-tumor spheroids as compared with that of PTX liposome and PTX nanoparticle. Accumulated evidence has revealed the overexpression of LDLR in breast cancer cells and the significantly variable expression profiles among different breast cancer cell subtypes [12]. Compared with estrogen receptor-positive breast cancer cells, LDLR was reportedly expressed at a higher level in a typical TNBC cell line, which in turn, could accelerate the aggressive and metastatic ability of TNBC cells by taking up, storing, and utilizing exogenous LDL-cholesterol mediated by LDLR [45]. The improved intracellular delivery, deep penetration, and extensive distribution of PTX emulsion in TNBC tumor spheroids through the LDLR-mediated internalization pathway may account for the superior apoptosis-inducing effect and enhanced tumor spheroid growth inhibition observed in the present study.

Although PTX emulsion exhibited superior in vitro antitumor effect compared to PTX liposome and PTX nanoparticle on 4T1 cells, no significant differences in the in vivo antitumor efficacy were found among the three types of PTX nanoformulations in immunocompetent orthotopic 4T1 murine TNBC model (Fig. 6). As TNBC is a subtype of breast cancer with high metastatic ability, a comprehensive evaluation of antitumor and antitumor metastasis efficacy may better reveal the clinical potential of different PTX nanoformulations in the treatment of TNBC. The comparative results of the in vivo antitumor efficacy obtained in the present study were inconsistent with those reported previously. A previous study revealed that PTX nanoparticle exhibited increased antitumor efficacy and improved therapeutic index in multiple human tumor xenograft models compared with an equitoxic dose of PTX injection, which may be attributed to the more effective intratumoral accumulation of PTX [27]. PTX emulsion has also been validated in terms of their superior in vivo antitumor efficacy and safety in nude mice bearing human TNBC xenografts, in comparison to PTX injection at the MTD [11]. The reasons for these contradictory outcomes are manifold, with the most critical being that the immune status of the tumor animal models used is strikingly different. Immune-deficient tumor-bearing mice were used in the aforementioned preclinical studies, whereas the antitumor efficacy of the present study was evaluated in immunocompetent normal mice. The immune system has critical roles in both cancer progression and regression, and the success of cancer treatment is closely related to the status of host immunity [17, 18]. Although MTD chemotherapy can appreciably eradicate cancer cells in a relatively short time, the high dose is also toxic to immune cells, including T cells and DCs, leading to lymphopenia and immunosuppression [20–23, 39–41, 46, 47]. Additionally, conventional MTD chemotherapy-induced lymphopenia has been associated with a worse prognosis and supposedly reflects the immunosuppressive state, which adversely influences the antitumor effect and may partly explain the failure of adjuvant chemotherapy [21]. Thus, the MTD nanomedicines–host immune system interactions, which were neglected in most preclinical studies due to immunodeficiency in nude mice, may have a significant impact on the antitumor efficacy of nanomedicines in immunocompetent mouse models. In the present study, the immunosuppressive effect of PTX nanoformulations on lymphocytes in the MTD regimen was investigated in immunocompetent mouse models. As shown in Figs. 8, 9, the immune profile (T cells and DCs) of mice following MTD chemotherapy treatment demonstrated that the MTD regimen of all PTX nanoformulations significantly depleted CD3+ and DCs and reduced the percentage of CD4+ or CD8+ in CD3+ cells, as well as significantly suppressed DCs maturation. There is increasing evidence that the circulating lymphocytes play a central role in the antitumor effect and chemotherapy-related lymphopenia and immunosuppression are associated with poor outcomes. [21, 24, 25] Thus, the MTD nanomedicines-induced immunosuppressive effect may weaken the lymphocyte-mediated antitumor cellular immune response and may partly account for the lack of differences in the in vivo antitumor outcome between PTX injection and nanoformulations in present study [21, 48]. For this reason, low-dose, frequent, and regular administration of chemotherapy referred to as metronomic chemotherapy, which can preserve the major immune cells and maintain the corresponding antitumor immunity, has gradually become an emerging alternative to conventional MTD chemotherapy [46, 49–51]. Moreover, host immune status is criticle for immunotherapies that are in development, and careful consideration of sequencing and dose may be required if these are to be combined with cytotoxic chemotherapies.

## Conclusions

Compared with PTX liposome and PTX nanoparticle, PTX emulsion was superior in inducing apoptosis and had a markedly more pronounced inhibitory effect on 4T1-tumor spheroids. However, no significant differences in the in vivo antitumor efficacy were found among the three types of PTX nanoformulations in the immunocompetent orthotopic 4T1 murine TNBC model. The MTD PTX nanomedicines-induced lymphopenia and immunosuppression may be the possible mechanism for the lack of differences in the in vivo antitumor outcome of PTX nanoformulations.

## Data Availability

The datasets used and/or analyzed during the current study are available from the corresponding author on reasonable request.
